# PD-1 gene rs10204525 and rs7421861 polymorphisms are associated with increased risk and clinical features of esophageal cancer in a Chinese Han population

**DOI:** 10.18632/aging.102845

**Published:** 2020-02-21

**Authors:** Bao Zang, Chen Chen, Jian-Qiang Zhao

**Affiliations:** 1 Department of Thoracic Surgery, The Affiliated Huaian No.1 People’s Hospital of Nanjing Medical University, Huaian, Jiangsu, China

**Keywords:** PD-1, esophageal cancer, case-control study

## Abstract

*Programmed death-1* (*PD-1*) polymorphisms have been associated with esophageal cancer risk. Here, the aims of this case-control study were to explore whether three *PD-1* polymorphisms (rs10204525, rs7421861, and rs36084323) were related with the risk and clinical features of esophageal cancer in Chinese Han subjects (n = 814 cases and 961 controls). We found that rs10204525 and rs7421861, but not rs36084323, conferred increased susceptibility to esophageal cancer. Subgroup analysis revealed that all three loci increased the risk of esophageal cancer among men, and that rs10204525 and rs7421861 correlated with increased risk among patients ≥ 60 years old. The rs10204525 and rs7421861 polymorphisms were associated with higher TNM stage, and rs10204525 was associated with distant metastasis. The combination of smoking and either the rs10204525 or rs7421861 genotype conferred an increased risk to esophageal cancer, which is indicative of potential gene-environment interactions. The rs10204525 and rs7421861 polymorphisms correlated with increased PD-1 gene and protein levels, and Kaplan-Meier survival curves showed higher *PD-1* gene expression was related to poorer overall survival. These data indicate the rs10204525 and rs7421861 polymorphisms of *PD-1* gene confer an increased risk of esophageal cancer among Chinese Han individuals.

## INTRODUCTION

Esophageal cancer is the 8^th^ most common tumor and the 6^th^ cause of tumor associated death in the world [1]. The 5-year survival rate of this disorder is only 15–25%, owing to the aggressive nature of the disease and resistance to therapy. Approximately 50% of esophageal cancer patients worldwide occur in China. Patients typically present with progressive dysphagia when diagnosed [2]. Esophageal cancer can arise in the upper (<10% of cases), middle (>50%), or lower (>20%) segment of the esophagus [2, 3]. Patients in high-risk areas in China therefore undergo routine endoscopic screening, which is useful for early diagnosis and effective treatment of esophageal cancer.

Surgical or endoscopic resection and chemoradiotherapy are the standard treatments for esophageal cancer, although the therapeutic strategy varies according to stage [4, 5]. Many patients who present with resectable esophageal cancer ultimately develop local recurrence and metastasis [6]. These patients are primarily managed with chemotherapy (e.g. fluoropyrimidine/platinum) [7]. Additional chemotherapy methods include capecitabine, S-1, infusional 5-fluorouracil (5-FU) and other 5-FU pro-drugs, and oxaliplatin or cisplatin [7]. The response rates to 5-FU in combination with cisplatin were reported to be 30–40% and 40–50% for esophageal adenocarcinoma and esophageal squamous cell carcinoma (ESCC), respectively [6]. In total, improvements of chemotherapy were disappointing over the last 3 decades, and current efforts should pay attention to targeted therapies and immunotherapy. Several new drugs such as ramucirumab, capecitabine, oxaliplatin, and everolimus have also demonstrated therapeutic efficacy in esophageal cancer [6, 8–12]. However, some patients have developed resistance to these therapies, and the underlying mechanisms were complex and poorly understood. Drug resistance may in part be mediated by the long non-coding RNA CCAT1 [13]. Additionally, microRNA-10b and CDKN3 have been shown to contribute to cisplatin resistance [14, 15].

Esophageal cancer contains 2 primary pathological subtypes: adenocarcinoma and ESCC. Similar genetic and environmental risk factors for esophageal adenocarcinoma and ESCC have been identified including gender, race, obesity, nutrition, smoking, and alcohol consumption [1]. Interestingly, polymorphisms in the *programmed death-1* (*PD-1*) gene (located in chromosome 2q37.3) have been associated with esophageal cancer risk and prognosis [16–18].

PD-1, a 50–55 kDa type I transmembrane glycoprotein, was first isolated from activated T cells [19, 20]. It consists of a transmembrane domain, extracellular domain, and intracellular region. It is reported to express on the surface of some immune cells including activated monocytes, T cells, natural killer cells, B cells, and NK T cells [21, 22]. PD-1 negatively regulated the regulatory and effector T cells. It plays a critical role in suppressing the immune response to promote self-tolerance [23]. However, by suppressing the immune response, it can contribute to cancer progression. PD-1 gene expression was associated with T cell activation-induced apoptosis in murine T cell hybridomas [24]. Additionally, persistent PD-1 expression in tumor-infiltrating lymphocytes was related with poor prognosis and cancer recurrence [25]. Two FDA-approved monoclonal antibodies targeting PD-1 including pembrolizumab and nivolumab have demonstrated efficacy for several cancer treatment [26, 27]. Anti-PD-1 therapy promoted regression of advanced tumors and improved survival, particularly among subsets of patients with solid tumors, and demonstrated durable effects and tolerable toxicities [27]. Biomarkers including density of tumor infiltrating lymphocyte, mismatch-repair deficiency, PD-L1 expression, and tumor mutational burden, predicted treatment effect of anti-PD-1 therapy [26].

*PD-1* gene polymorphisms were associated with the risk of various cancers [1, 28–30]. Beyond foregoing disorders, PD-1 is overexpressed in different cancers, including esophageal cancer. For example, the rs2227981, rs2227982, and rs3608432 polymorphisms were related to lung adenocarcinoma risk and prognosis [31]. However, no associations were observed in basal cell carcinoma [32]. The *PD-1*.5 C/T polymorphism increased the risk of cervical [33], lung [34], gastric [35], colon [36], thyroid cancers [37]. The rs2227982 C>T polymorphism associated with gastric cardia adenocarcinoma risk [38]. Interestingly, it was reduced the risk of breast cancer [39] and increased the risk of ovarian cancer [40]. Hua et al. mentioned that *PD-1* gene polymorphisms may regulate the breast cancer susceptibility and prognosis in Chinese individuals [41], while inconsistent findings were obtained in the study by Haghshenas et al. [42]. Thus, several meta-analyses were conducted to solve these conflicting findings [43–45]. Data summarized that *PD-1* rs11568821 and rs2227981 polymorphisms decreased the overall cancer risk, and *PD-1* rs7421861 polymorphism was associated with an increased risk of overall cancer [43]. No significant association between some SNPs (rs2227982, rs10204525, rs36084323, and rs2890658 polymorphisms) and overall cancer risk was obtained [43]. To be honest, these loci of *PD-1* gene might be potential biomarkers for predicting susceptibility to cancers and therapeutic markers for cancer treatment.

Considering the vital role of *PD-1* gene polymorphisms in cancers, we designed this hospital-based case-control study containing 814 esophageal cancer patients and 961 healthy controls. The aims of this case-control study were to investigate whether three *PD-1* gene polymorphisms (rs10204525, rs36084323, and rs7421861) were related with esophageal cancer risk and clinical features in Chinese subjects.

## RESULTS

### Population characteristics

We performed a case-control study consisting of 814 esophageal cancer patients and 961 age- and gender-matched controls. The baseline characteristics of these patients including TNM stage, pathological grade, and distant metastases are shown in Table 1. The mean ages of the controls and cases were 60.91 and 60.66years, respectively. No differences were observed in smoking or alcohol between the two groups. The majority of the patients (85%) were diagnosed with ESCC.

**Table 1:** Patient demographics and risk factors in esophageal cancer.

**Characteristics**	**Case (N=814)**	**Control (N=961)**	***P***
Age	60.66 (36-82)	60.91 (38-85)	0.495
Sex			0.440
Male	577(70.9%)	665(69.2%)	
Female	237(29.1%)	296(30.8%)	
Smoking			0.771
YES	430(52.8%)	501(52.1%)	
NO	384(47.2%)	460(47.9%)	
Alcohol			0.358
YES	470 (57.7%)	534(55.6%)	
NO	344(42.3%)	427(44.4%)	
TNM stage			
I+II	424(52.1%)		
III+IV	390(47.9%)		
Pathological grading			
Well differentiation	320(39.3%)		
Moderately differentiation	378(46.4%)		
Poorly differentiation	116(14.3%)		
Histology			
Squamous cell carcinoma	782(96.1%)		
Others	32(3.9%)		
Distant metastasis			
M0	723(88.8%)		
M1	91(11.2%)		

TNM stage = Tumor node metastasis stage

### PD-1 polymorphisms increase the risk of esophageal cancer

We evaluated the associations between three polymorphisms in *PD-1* (rs10204525, rs7421861, and rs36084323) and the risk of esophageal cancer. The distributions of the genotypes of the *PD-1* variants among the case and control populations are presented in Table 2 and Supplementary Figure 1. The GG genotype of rs10204525 polymorphism increased the risk of esophageal cancer compared to the more common AA genotype (GG vs. AA: adjusted odds ratio [OR] = 1.65, 95% confidence interval [CI] = 1.12–2.45; *P* = 0.012). This association was significant under recessive, dominant, and allelic models. The TT genotype of rs7421861 was related with a 1.45-fold higher risk of esophageal cancer compared to the CC genotype (TT vs. CC: OR = 1.45, 95% CI = 1.06–1.99); *P* = 0.022). We did not observe an association between rs36084323 polymorphism and esophageal cancer risk.

**Table 2:** Genotype frequencies of PD-1 gene polymorphisms in cases and controls.

**Models**	**Genotype**	**Case (n, %)^a^**	**Control (n, %)^a^**	**OR (95% CI)**	***P*-value**	**P (FDR)^b^**	**OR (95% CI)^c^**	***P*-value^c^**
**rs10204525**								
Co-dominant	AA	420(51.7%)	551(57.4%)	1.00(reference)				
Heterozygote	AG	329(40.5%)	359(37.4%)	1.20(0.99-1.46)	0.066		1.20(0.99-1.46)	0.070
Homozygote	GG	63(7.8%)	50(5.2%)	**1.65(1.12-2.45)**	**0.012**		**1.67(1.13-2.48)**	**0.010**
Dominant	AA	420(51.7%)	551(57.4%)	1.00(reference)				
	GG+AG	392(48.3%)	409(42.6%)	**1.26(1.04-1.52)**	**0.017**		**1.26(1.04-1.52)**	**0.017**
Recessive	AG+AA	749(92.2%)	910(94.8%)	1.00(reference)				
	GG	63(7.8%)	50(5.2%)	**1.53(1.04-2.25)**	**0.030**		**1.55(1.06,2.28)**	**0.025**
Allele	A	1169(72.0%)	1461(76.1%)	1.00(reference)				
	G	455(28.0%)	459(23.9%)	**1.24(1.07-1.44)**	**0.005**	**0.015**		
**rs36084323**								
Co-dominant	GG	673(82.8%)	761(79.2%)	1.00(reference)				
Heterozygote	GA	132(16.2%)	188(19.6%)	0.79(0.62-1.02)	0.066		0.79(0.62-1.01)	0.064
Homozygote	AA	8(1.0%)	12(1.2%)	0.75(0.31-1.86)	0.539		0.75(0.30-1.85)	0.530
Dominant	GG	673(82.8%)	761(79.2%)	1.00(reference)				
	AA+GA	140(17.2%)	200(20.8%)	0.79(0.62-1.01)	0.056		0.79(0.62-1.00)	0.054
Recessive	GA+GG	805(99.0%)	949(98.8%)	1.00(reference)				
	AA	8(1.0%)	12(1.2%)	0.79(0.32-1.93)	0.600		0.78(0.32,1.92)	0.589
Allele	G	1478(90.9%)	1710(89.0%)	1.00(reference)				
	A	148(9.1%)	212(11.0%)	0.81(0.65-1.01)	0.058	0.058		
**rs7421861**								
Co-dominant	CC	343(42.2%)	457(47.6%)	1.00(reference)	-		-	-
Heterozygote	CT	370(45.5%)	411(42.8%)	1.19(0.98-1.46)	0.072		1.20(0.98-1.46)	0.074
Homozygote	TT	100(12.3%)	92(9.6%)	**1.45(1.06-1.99)**	**0.022**		**1.44(1.05,1.97)**	**0.024**
Dominant	CC	343(42.2%)	457(47.6%)	1.00(reference)	-		-	-
	TT+CT	470(57.8%)	503(52.4%)	**1.25(1.03-1.50)**	**0.023**		**1.24(1.03,1.50)**	**0.024**
Recessive	CT+CC	713(87.7%)	868(90.4%)	1.00(reference)	-		-	-
	TT	100(12.3%)	92(9.6%)	1.32(0.98-1.79)	0.067		1.32(0.97,1.78)	0.074
Allele	C	1056(64.9%)	1325(69.0%)	1.00(reference)	-		-	-
	T	570(35.1%)	595(31.0%)	**1.20(1.04-1.38)**	**0.010**	**0.015**		

^a^The genotyping was successful in 812 cases and 960 controls for rs10204525; The genotyping was successful in 813 cases and 961 controls for rs36084323; The genotyping was successful in 813 cases and 960 controls for rs7421861.

Bold values are statistically significant (P <0.05).

^b^P (FDR) values were calculated with for false discovery rate (FDR) and P < 0.05/3 was considered significant.

^c^Adjust for sex, age, smoking and drinking.

We next stratified patients by age, gender, pathological subtype, alcohol consumption, and smoking. The genotype numbers among different subgroups are summarized in Supplementary Figure 2. The association between rs10204525 polymorphism and the esophageal cancer risk was stronger among men, those who smoked or consumed alcohol, and those ≥ 60 years old (Table 3). The rs7421861 polymorphism demonstrated a significant association with esophageal cancer risk among men and among smokers (Table 3). The rs36084323 polymorphism was only related to esophageal cancer risk among men. Finally, rs10204525 and rs7421861 polymorphisms increased the risk of ESCC (Supplementary Tables 1 and 2).

**Table 3:** Stratified analyses between PD-1 gene polymorphisms and the risk of esophageal cancer.

**Variable**	**Genotypes (case/control)**			**Heterozygous model**	**Homozygous model**	**Recessive model**	**Dominant model**
	**Wild**	**Heterozygote**	**Homozygous**				
**rs10204525**	**AA**	**AG**	**GG**	**AG vs. AA**	**GG vs. AA**	**GG vs. AA+AG**	**GG+AG vs. AA**
Sex							
Male	299/388	236/244	41/33	1.26(0.99-1.59); 0.057	1.61(0.99-2.61); 0.052	1.47(0.92-2.35); 0.112	**1.30(1.04-1.63); 0.023**
Female	121/163	93/115	22/17	1.09(0.76-1.56); 0.642	1.74(0.89-3.43); 0.107	1.68(0.87-3.25); 0.122	1.17(0.83-1.65); 0.361
Smoking							
Yes	222/290	168/185	38/26	1.19(0.90-1.56); 0.219	**1.91(1.13-3.24); 0.017**	**1.78(1.06-2.98); 0.029**	1.28(0.98-1.65); 0.066
No	198/261	161/174	25/24	1.22(0.92-1.62); 0.169	1.37(0.76-2.48); 0.292	1.26(0.71-2.25); 0.429	1.14(0.94-1.63); 0.124
Alcohol							
Yes	240/301	191/209	38/24	1.15(0.88,1.49); 0.303	**1.99(1.16,3.40); 0.013**	**1.87(1.11,3.17); 0.020**	1.23(0.96,1.58); 0.100
No	180/250	138/150	25/26	1.29(0.95,1.73); 0.110	1.34(0.75,2.39); 0.330	1.21(0.69,2.14); 0.512	1.29(0.97,1.71); 0.085
Age (years)							
<60	207/238	134/158	22/21	0.98(0.73,1.31); 0.868	1.21(0.64,2.26); 0.559	1.22(0.66,2.25); 0.531	1.00(0.75,1.33); 0.989
≥60	213/313	195/201	41/29	**1.43(1.10,1.85); 0.008**	**2.08(1.25,3.45); 0.005**	**1.78(1.09,2.92); 0.022**	**1.51(1.17,1.94); 0.001**
**rs36084323**	**GG**	**GA**	**AA**	**GA vs. GG**	**AA vs. GG**	**AA vs. GG+GA**	**AA+GA vs. GG**
Sex							
Male	484/520	87/135	6/10	**0.69(0.52-0.93); 0.015**	0.65(0.23-1.79); 0.399	0.69(0.25-1.91); 0.472	**0.69(0.52-0.92); 0.011**
Female	189/241	45/53	2/2	1.08(0.70-1.68); 0.724	1.28(0.78-9.14); 0.809	1.26(0.18-8.99); 0.820	1.09(0.71-1.68); 0.697
Smoking							
Yes	355/395	71/101	4/5	0.78(0.56-1.09); 0.152	0.89(0.24-3.34); 0.863	0.93(0.25-3.49); 0.916	0.79(0.57-1.09); 0.154
No	318/366	61/87	4/7	0.81(0.56-1.16); 0.243	0.66(0.19-2.27); 0.507	0.68(0.20-2.35); 0.546	0.80(0.56-1.13); 0.201
Alcohol							
Yes	388/417	75/111	6/6	0.73(0.53,1.00); 0.053	1.08(0.34,3.36); 0.901	1.14(0.37,3.56); 0.821	0.86(0.59,1.24); 0.416
No	285/344	57/77	2/6	0.89(0.61,1.30); 0.558	0.40(0.08,2.01); 0.268	0.41(0.08,2.05); 0.278	0.74(0.54,1.02); 0.307
Age (years)							
<60	300/322	58/86	5/10	0.72(0.50,1.05); 0.086	0.54(0.18,1.59); 0.261	0.57(0.19,1.68); 0.309	0.70(0.49,1.00); 0.053
≥60	173/439	74/102	3/2	0.85(0.61,1.19); 0.348	**1.77(0.29,10.62); 0.535**	1.81(0.30,10.90); 0.515	0.87(0.63,1.21); 0.407
**rs7421861**	**CC**	**CT**	**TT**	**CT vs. CC**	**TT vs. CC**	**TT vs. CC+CT**	**TT+CT vs. CC**
Sex							
Male	233/318	272/283	71/63	**1.31(1.04-1.66); 0.025**	**1.54(1.05-2.25); 0.026**	1.34(0.94-1.92); 0.109	**1.35(1.08-1.70); 0.009**
Female	110/139	98/128	29/29	0.97(0.67-1.39); 0.858	1.26(0.71-2.24); 0.423	1.28(0.74-2.22); 0.370	1.02(0.73-1.44); 0.900
Smoking							
Yes	174/240	195/221	61/39	1.22(0.93-1.60); 0.160	**2.16(1.38-3.37); 0.001**	**1.95(1.28-2.99); 0.002**	**1.36(1.05-1.76); 0.021**
No	169/217	175/190	39/53	1.18(0.89-1.58); 0.253	0.95(0.60-1.50); 0.809	0.87(0.56-1.35); 0.535	1.13(0.86-1.49); 0.377
Alcohol							
Yes	191/261	222/225	44/45	0.73(0.53,1.00); 0.053	1.08(0.34,3.36); 0.901	1.14(0.37,3.56); 0.821	0.86(0.59,1.24); 0.416
No	152/196	148/186	56/47143	0.89(0.61,1.30); 0.558	0.40(0.08,2.01); 0.268	0.41(0.08,2.05); 0.278	0.74(0.54,1.02); 0.307
Age (years)							
<60	143/205	173/170	47/42	0.72(0.50,1.05); 0.086	0.54(0.18,1.59); 0.261	0.57(0.19,1.68); 0.309	0.70(0.49,1.00); 0.053
≥60	200/252	197/241	53/50	0.85(0.61,1.19); 0.348	1.77(0.29,10.62); 0.535	1.81(0.30,10.90); 0.515	0.87(0.63,1.21); 0.407

Bold values are statistically significant (*P* <0.05).

### Cross-over analysis

We next analyzed the joint effects of the *PD-1* polymorphisms and either smoking or alcohol consumption on esophageal cancer risk (Table 4). The GG genotype of rs10204525 did not confer an increased risk to esophageal cancer. Additionally, smoking had no association with the risk of esophageal cancer. However, smokers with the GG genotype of rs10204525 polymorphism showed an increased risk of esophageal cancer compared to non-smokers with the AA genotype (OR = 1.93, 95% CI = 1.13–3.28; *P* = 0.014). These data indicate that there is a strong interaction between the GG genotype of rs10204525 and smoking. The TT genotype of rs7421861 was also not associated with an increased risk of esophageal cancer. However, smokers with the TT genotype of rs7421861 had a significantly increased risk of esophageal cancer. No interaction between rs36084323 and either smoking or alcohol consumption was observed.

**Table 4:** Genetic (G) and environmental (E) factors 2*4 fork analysis.

**G^a^**	**E^b^**	**Case**	**Control**	**OR (95%CI); P value**	**Reflecting information**
**rs10204525**					
GG vs. AA	Smoking				
+	+	38	26	**1.93(1.13,3.28); 0.014**	G, E combined effect
+	-	25	24	1.37(0.76,2.48); 0.291	G alone effect
-	+	222	290	1.01(0.78,1.30); 0.944	E alone effect
-	-	198	261	1.00 (reference)	Common control
AG vs. AA	Smoking				
+	+	168	185	1.20(0.91,1.58); 0.206	G, E combined effect
+	-	161	174	1.22(0.92,1.62); 0.169	G alone effect
-	+	222	290	1.01(0.78,1.30); 0.944	E alone effect
-	-	198	261	1.00 (reference)	Common control
GG vs. AA	Drinking				
+	+	38	50	1.06(0.66,1.68); 0.819	G, E combined effect
+	-	25	24	1.45(0.80,2.62); 0.220	G alone effect
-	+	240	301	1.11(0.86,1.43); 0.434	E alone effect
-	-	180	250	1.00 (reference)	Common control
AG vs. AA	Drinking				
+	+	191	209	1.27(0.97,1.67); 0.088	G, E combined effect
+	-	138	150	1.28(0.95,1.73); 0.109	G alone effect
-	+	240	301	1.11(0.86,1.43); 0.434	E alone effect
-	-	180	250	1.00 (reference)	Common control
**rs36084323**					
AA vs. GG	Smoking				
+	+	4	5	0.99(0.27,3.73); 0.993	G, E combined effect
+	-	4	7	0.71(0.21,2.45); 0.585	G alone effect
-	+	355	366	1.21(0.98,1.48); 0.079	E alone effect
-	-	318	395	1.00 (reference)	Common control
GA vs. GG	Smoking				
+	+	71	101	0.87(0.62,1.22); 0.431	G, E combined effect
+	-	61	87	0.87(0.61,1.25); 0.450	G alone effect
-	+	355	366	1.21(0.98,1.48); 0.079	E alone effect
-	-	318	395	1.00 (reference)	Common control
AA vs. GG	Drinking				
+	+	6	6	1.21(0.39,3.78); 0.747	G, E combined effect
+	-	2	6	0.40(0.08,2.01); 0.251	G alone effect
-	+	388	417	1.12(0.91,1.38); 0.277	E alone effect
-	-	285	344	1.00 (reference)	Common control
GA vs. GG	Drinking				
+	+	75	111	0.82(0.59,1.14); 0.229	G, E combined effect
+	-	57	77	0.89(0.61,1.30); 0.558	G alone effect
-	+	388	417	1.12(0.91,1.38); 0.277	E alone effect
-	-	285	344	1.00 (reference)	Common control
**Rs7421861**					
TT vs. CC	Smoking				
+	+	61	39	**2.01(1.28,3.15); 0.002**	G, E combined effect
+	-	39	53	0.95(0.60,1.50); 0.809	G alone effect
-	+	174	240	0.93(0.70,1.23); 0.617	E alone effect
-	-	169	217	1.00 (reference)	Common control
CT vs. CC	Smoking				
+	+	195	221	1.13(0.86,1.50); 0.379	G, E combined effect
+	-	175	190	1.18(0.89,1.58); 0.252	G alone effect
-	+	174	240	0.93(0.70,1.23); 0.617	E alone effect
-	-	169	217	1.00 (reference)	Common control
TT vs. CC	Drinking				
+	+	56	47	1.54(0.99,2.39); 0.056	G, E combined effect
+	-	44	45	1.26(0.79,2.01); 0.330	G alone effect
-	+	191	261	0.94(0.71,1.25); 0.687	E alone effect
-	-	152	196	1.00 (reference)	Common control
CT vs. CC	Drinking				
+	+	222	225	1.27(0.96,1.69); 0.093	G, E combined effect
+	-	148	186	1.03(0.76,1.39); 0.868	G alone effect
-	+	191	261	0.94(0.71,1.25); 0.687	E alone effect
-	-	152	196	1.00 (reference)	Common control

^a^G (+): PD-1 gene rs10204525/rs36084323/rs7421861 variants (Heterozygous or homozygous); G (-): wild type

^b^E(+): smoking/non-smoking; E(-): non-smoking/non-drinking

### *PD-1* gene polymorphisms correlate with the clinical features of esophageal cancer patients

We next investigated the relationship between *PD-1* gene polymorphisms and the clinical characteristics of esophageal cancer patients (Table 5). The GG genotype of rs10204525 polymorphism increased the risk of distant metastasis (OR = 2.21, 95% CI = 1.16–4.23; *P* = 0.014) and higher TNM stage (OR = 1.81, 95% CI = 1.05–3.12; *P* = 0.032). The AG genotype of rs10204525 was related to an increased risk of ESCC (OR = 1.61, 95% CI = 1.05–2.46; *P* = 0.029). Finally, the TT genotype of rs7421861was associated with higher TNM stage.

**Table 5:** The associations between PD-1 gene polymorphisms and clinical characteristics of esophageal cancer.

**Characteristics**	**Genotype distributions**			
**rs10204525**	**AA**	**AG**	**GG**	**AG+GG**
Pathological grading				
MD/WD	212//156	139/135	26/28	165/163
OR (95%CI); *P*-value	1.0 (reference)	0.76(0.55-1.04); 0.083	0.68(0.39-1.21); 0.190	0.75(0.55-1.01); 0.054
Pathological grading				
PD/WD	52/156	55/135	9/28	64/163
OR (95%CI); *P*-value	1.0 (reference)	1.22(0.78-1.91); 0.375	0.96(0.43-2.18); 0.930	1.18(0.77-1.81); 0.452
Distant metastasis				
M1/M0	52/368	60/269	15/48	75/317
OR (95%CI); *P*-value	1.0 (reference)	**1.58(1.06-2.36); 0.026**	**2.21(1.16-4.23); 0.014**	**1.67(1.14-2.46); 0.008**
Tumor node metastasis stage				
T3+T4 / T1+T2	206/214	153/176	40/23	193/199
OR (95%CI); *P*-value	1.0 (reference)	0.90(0.68-1.21); 0.489	**1.81(1.05-3.12); 0.032**	1.01(0.77-1.33); 0.958
Histology				
Squamous/Not Squamous	349/71	292/37	49/14	341/51
OR (95%CI); *P*-value	1.0 (reference)	**1.61(1.05-2.46); 0.029**	0.71(0.37-1.36); 0.301	1.36(0.92-2.01); 0.121
**rs36084323**	**GG**	**GA**	**AA**	**GA+AA**
Pathological grading				
MD/WD	321/268	60/49	5/3	65/52
OR (95%CI); *P*-value	1.0 (reference)	1.02(0.68-1.54); 0.916	1.39(0.33-5.88); 0.652	1.04(0.70-1.56); 0.834
Pathological grading				
PD/WD	93/268	23/49	½	13/52
OR (95%CI); *P*-value	1.0 (reference)	1.35(0.78-2.34); 0.279	1.44(0.13-16.08); 0.765	0.72(0.38-1.38); 0.323
Distant metastasis				
M1/M0	80/593	10/122	2/6	10/130
OR (95%CI); *P*-value	1.0 (reference)	0.61(0.31-1.21); 0.151	2.47(0.49-12.45); 0.257	0.57(0.29-1.13); 0.104
Tumor node metastasis stage				
T3+T4 / T1+T2	320/353	67/65	4/4	71/69
OR (95%CI); *P*-value	1.0 (reference)	1.14(0.78-1.65); 0.500	1.10(0.27-4.45); 0.890	1.14(0.79-1.63); 0.495
Histology				
Squamous/Not Squamous	569/104	115/17	7/1	122/18
OR (95%CI); *P*-value	1.0 (reference)	1.24(0.71-2.14); 0.449	1.28(0.16-10.51); 0.818	1.24(0.72-2.12); 0.434
**rs7421861**	**CC**	**CT**	**TT**	**CT+TT**
Pathological grading				
MD/WD	157/136	175/149	46/34	221/183
OR (95%CI); *P*-value	1.0 (reference)	1.02(0.74-1.40); 0.915	1.17(0.71-1.93); 0.533	1.05(0.77-1.42); 0.770
Pathological grading				
PD/WD	50/136	46/149	20/34	66/183
OR (95%CI); *P*-value	1.0 (reference)	0.84(0.53-1.33); 0.459	1.60(0.84-3.04); 0.148	0.98(0.64-1.51); 0.930
Distant metastasis				
M1/M0	44/299	41/329	6/94	47/423
OR (95%CI); *P*-value	1.0 (reference)	0.85(0.54-1.33); 0.472	0.43(0.18-1.05); 0.058	0.76(0.49-1.17); 0.207
Tumor node metastasis stage				
T3+T4 / T1+T2	149/194	185/185	56/44	241/229
OR (95%CI); *P*-value	1.0 (reference)	1.30(0.97-1.75); 0.079	**1.66(1.06-2.60); 0.027**	**1.37(1.04-1.81); 0.027**
Histology				
Squamous/Not Squamous	296/47	314/56	81/19	395/75
OR (95%CI); *P*-value	1.0 (reference)	0.89(0.59-1.35); 0.587	0.68(0.38-1.22); 0.191	0.84(0.56-1.24); 0.374

Bold values are statistically significant (*P* <0.05). PD = Poorly differentiation, MD= Moderately differentiation, WD= Well differentiation

### *PD-1* polymorphisms are related with PD-1 levels and prognosis among esophageal cancer patients

**Figure 1:**
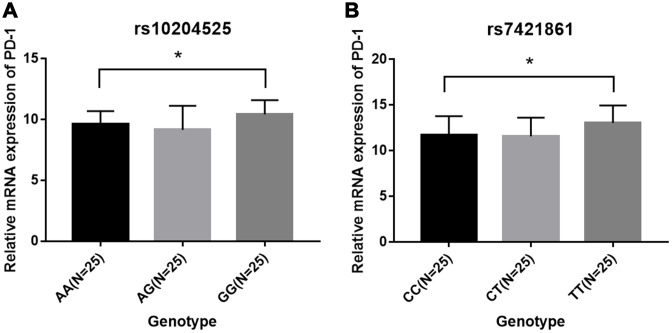
**Relative PD-1 mRNA expression among patients in each genotype group.** (**A**) rs10204525; (**B**) rs7421861.

**Figure 2:**
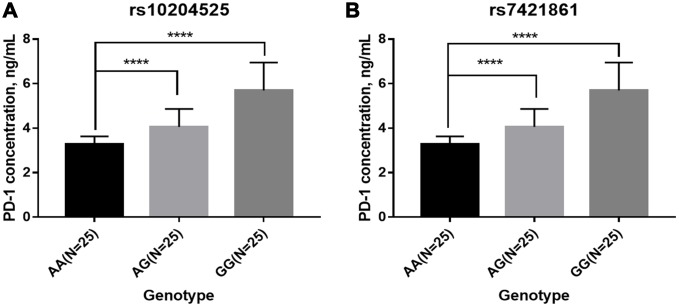
**Plasma PD-1 levels among patients in each genotype group.** (**A**) rs10204525; (**B**) rs7421861.

**Figure 3:**
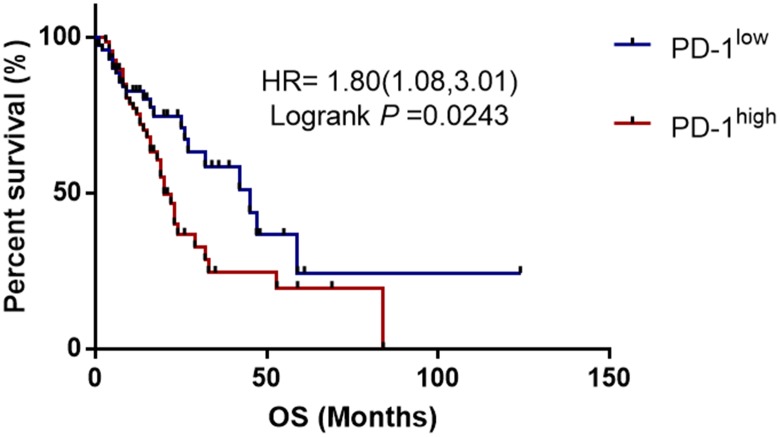
**Kaplan-Meier survival curves demonstrating the association between PD-1 expression and overall survival among esophageal cancer patients.**

The PD-1 expression levels were measured by qRT-PCR and ELISA in 150 esophageal cancer patients with different genotypes of rs10204525 and rs7421861 polymorphisms. Results showed PD-1 expression levels are significantly higher in GG genotype versus AA genotype carriers (*P* = 0.013; Figure 1). Similar findings were obtained for the rs7421861 polymorphism (*P* = 0.024). Similarly, increased PD-1 plasma levels are shown by Elisa (Figure 2). Moreover, higher PD-1 expressions showed worse overall survival among esophageal cancer patients (Figure 3). Thus, the mutant genotype of rs10204525 or rs7421861 polymorphism might contribute to worse survival of esophageal cancer patients by increasing the PD-1 expression.

## DISCUSSION

In this study, we investigated the relationship between *PD-1* gene variants and the risk of esophageal cancer and found *PD-1* gene rs7421861 and rs10204525 polymorphisms increased the risk of esophageal cancer in Chinese individuals. The combination of smoking and these genotypes showed a significantly higher risk for esophageal cancer. In addition, genotypes of rs10204525 and rs7421861 polymorphisms were shown to be associated with increased PD-1 gene and protein levels. Furthermore, higher PD-1 expressions were correlated with worse survival of esophageal cancer patients.

Increased expression of programmed death-ligand 1 (PD-L1) has frequently been observed in the brain, gastrointestinal tract, lung, liver, colorectum, kidney, pancreas, ovary, bladder, and esophagus cancers [46–49]. Monoclonal antibodies targeting PD-1 block the interaction with PD-L1 and have demonstrated efficacy in various malignancies [50]. PD-L1 expression was associated with clinical features in esophageal cancer and kidney clear cell carcinoma [51, 52]. Overexpression of PD-1 was also observed in hepatocellular carcinoma and adjacent tissue, and was correlated with the rs10204525 polymorphism in PD-1 [53].

The relationship between *PD-1* gene loci and cancer risk has been extensively studied, but the conclusions are inconsistent. As reported, *PD-1* gene rs2227982 C>T polymorphism other than rs10204525 A>G or rs7421861 T>C polymorphism was associated with gastric cardia adenocarcinoma [38]. PD-1 was highly expressed on liver cancer tissues and adjacent tissues and the PD-1 level was remarkably associated with *PD-1* gene rs10204525 polymorphism [53]. Yeo et al. showed PD-L1 expression was unregulated in lung squamous cell carcinoma [54]. Moreover, the *PD-L1* 8923 A/C polymorphism [55] and *PD-1*.5 C/T polymorphism [34] are risky factors of non-small cell lung cancer (NSCLC). Another study observed a significant link between the *PD-1* gene rs2227982 polymorphism and breast cancer risk in northwest Chinese women [39]. A meta-analysis concluded the *PD-1* gene rs36084323 polymorphism decreased cancer risk among Asians [44].

The AA genotype of rs10204525 in *PD-1* gene was previously associated with an increased risk of esophageal cancer and proposed to be a predictive biomarker for ESCC [17]. The rs36084323 T>C polymorphism previously reduced the risk of esophagogastric junction adenocarcinoma (EGJA) while the rs7421861 polymorphism increased the risk of EGJA in Chinese subjects [18]. In addition, the *PD-1* T_rs10204525_G_rs2227982_C_rs36084323_A_rs7421861_ haplotype significantly associated with a decreased risk of EGJA [18]. The rs10204525 polymorphism was not related to ESCC risk in the full cohort. However, a stratified analysis demonstrated that it was associated with a reduced risk of ESCC among male and younger patients [16]. Here we found *PD-1* rs10204525 and rs7421861 polymorphisms, but not rs36084323, increased the risk for esophageal cancer, which are obviously inconsistent with the above studies. The conflicting findings may be attributed to some causes, such as clinical heterogeneity. The pathological types were different among these studies, as we studied several pathological types, rather than single cancer types such as EGJA [18] and ESCC [16]. Secondly, the sample sizes of these studies were diverse. Thirdly, living environment and diets may also affect the results.

Furthermore, stratified analyses of age, smoking, sex, and alcohol status were conducted. The rs10204525, rs36084323, rs7421861 polymorphisms increased the risk of esophageal cancer among men in our study. Subgroup analysis revealed that rs10204525 polymorphism elevated the risk of esophageal cancer among patients who consumed alcohol and among patients ≥ 60 years old. Cross-over analysis indicated that smoking in combination with either the rs10204525 or rs7421861 polymorphism significantly contributed to an increased risk of esophageal cancer.

Next, we explored the associations between *PD-1* gene polymorphisms and clinical features of esophageal cancer. It was found that rs7421861 and rs10204525 polymorphisms were associated with distant metastasis, and that rs7421861 was also associated with higher TNM stage. However, rs36084323 polymorphism was not associated with esophageal cancer metastasis. Interestingly, both the rs10204525 and rs7421861 polymorphisms were associated with higher PD-1 gene and plasma levels in esophageal cancer patients. Furthermore, Kaplan-Meier survival curves showed higher PD-1 gene expression contributed to worse survival of esophageal cancer patients. These results are in line with those of previous studies, which demonstrated associations between PD-L1 polymorphisms and poor prognosis [54] and survival [56] among lung cancer patients. Thus, we assumed *PD-1* gene variants increased the PD-1 gene levels, thereby contributing to esophageal cancer metastasis and worse survival.

This present study did have some potential limitations. First, the limited sample size of this study could not exactly uncover the relationship of the *PD-1* gene rs7421861, rs10204525, rs36084323 polymorphisms with esophageal cancer susceptibility. Second, the cases and controls were selected only from Chinese population. Different diet culture, living environment, habits and customs may also contribute to the development of this disorder and selection bias to the whole ethnic groups can still not be ignored. Third, further functional analyses were necessary to uncover how the *PD-1* gene polymorphisms affect esophageal cancer. Fourth, we could not perform related experiments to explore the underlying mechanisms by which the *PD-1* variants conferred an increased risk to esophageal cancer. Moreover, only three variants of *PD-1* gene were explored. Last, potential gene-gene or gene-environment interactions were not obtained.

In summary, the rs7421861 and rs10204525 polymorphisms in *PD-1* gene increase the risk of esophageal cancer in a Chinese Han population. These polymorphisms could be potential diagnostic and therapeutic biomarkers. However, the conclusions still need further validation by more studies with large sample sizes in other ethnicities.

## MATERIALS AND METHODS

### Subjects

814 patients with newly diagnosed esophageal cancer and 961 cancer-free controls were enrolled from the Affiliated Huai’an No.1 People’s Hospital of Nanjing Medical University. All patients were ≥ 18 years old with no history of other cancers. Patients with history of esophageal disease, a second primary tumor, or tumor of unknown origin were excluded. The controls consisted of individuals who received a comprehensive health examination and had no related history of cancer or autoimmune disease.

Patient demographics and other clinical data were collected using a written questionnaire. Smokers were regarded as smoking more than one cigarette per day for at least 1 year. Drinkers were defined as consumption of alcoholic beverages more than once a week for ≥ 1 year. The Institutional Review Board of Huai’an No.1 People’s Hospital approved this study. Written informed consent was got from all participants. Patient confidentiality was maintained according to the Helsinki declaration.

### DNA extraction and genotyping

Peripheral blood (2 mL) was collected from each patient following surgery. Patients were enrolled in the study if post-operative pathological analysis confirmed a diagnosed of esophageal cancer. Using a Puregene DNA Purification Kit, Genomic DNA was obtained from peripheral blood (Gentra, Minneapolis, MN, USA). DNA concentration and purity were analyzed by absorbance and gel electrophoresis, respectively. Genotyping was performed using matrix-assisted laser desorption/ionization time-of-flight mass spectrometry (MALDI-TOFMS) on a MassARRAY system (Sequenom, San Diego, CA, USA). Approximately 5% of the samples were randomly used for repeat assays and a 100% concordance rate was observed.

### Quantitative RT-PCR

Total RNA was isolated from peripheral venous blood using the Trizol reagent (Invitrogen, Carlsbad, CA, USA). RNA was reverse-transcribed into cDNA using the SuperScript^TM^ II Reverse Transcriptase (Invitrogen). Relative PD-1 expression was quantified by real-time PCR using TaqMan^®^ assays. Beta-actin was used as an internal reference. The forward and reverse primers were as follows: 5'-GCACGAGGGACAATAGGA-3', 5'-GAC AATGGTGGCATACT C-3' (PD-1); 5'-AGGTCGGTGT GAACGGATTTG-3', 5'-TGTAGACCATGTAGTTG AGGTCA-3' (GAPDH). Relative gene expression of PD-1 was calculated by the 2^-△△CT^ method.

### ELISA

PD-1 levels in plasma were evaluated using a human PD-1 ELISA kit (Sino Biological, Beijing, China). The absorbance was assessed by use of a Tecan Infinite F50 Absorbance Microplate Reader (Tecan, Männedorf, Switzerland). Plasma PD-1 levels were quantified using a standard curve.

### Kaplan-Meier survival analysis

The prognostic value of PD-1 mRNA expression in ESCC was assessed using OncoLnc (http://www. oncolnc.org), which contains survival data for 144 patients derived from The Cancer Genome Atlas dataset. Hazard ratios and 95% CIs were obtained using a Cox proportional-hazards model.

### Statistical analysis

All statistical analyses were performed using SPSS 13.0 (IBM, Armonk, NY, USA). Graphs were generated using GraphPad Prism 5 (GraphPad Software, La Jolla, CA, USA). The observed genotype frequencies in controls were calculated for Hardy-Weinberg equilibrium test using goodness-of-fit chi-square tests [57]. Categorical variables were assessed using χ^2^ tests and displayed as frequencies (percentages). Continuous variables in a normal distribution were evaluated using independent samples t-tests or one-way analysis of variance and expressed as the mean ± standard deviation. Logistic regression assuming allelic, dominant, recessive, and co-dominant models was performed to analyze the associations between the polymorphisms and disease risk. *P* values < 0.05 were considered significant [58, 59].

## Supplementary Material

Supplementary Figures

Supplementary Tables
