# Supplementary material for: PD-1 gene rs10204525 and rs7421861 polymorphisms are associated with increased risk and clinical features of esophageal cancer in a Chinese Han population
Source: Aging (Albany NY). 2020 Feb 21;12(4):3771–90. doi: 10.18632/aging.102845 (PMC7066885; doi:10.18632/aging.102845)
Supplement: Supplementary Figures [file aging-12-102845-s002..pdf]

SUPPLEMENTARY FIGURES

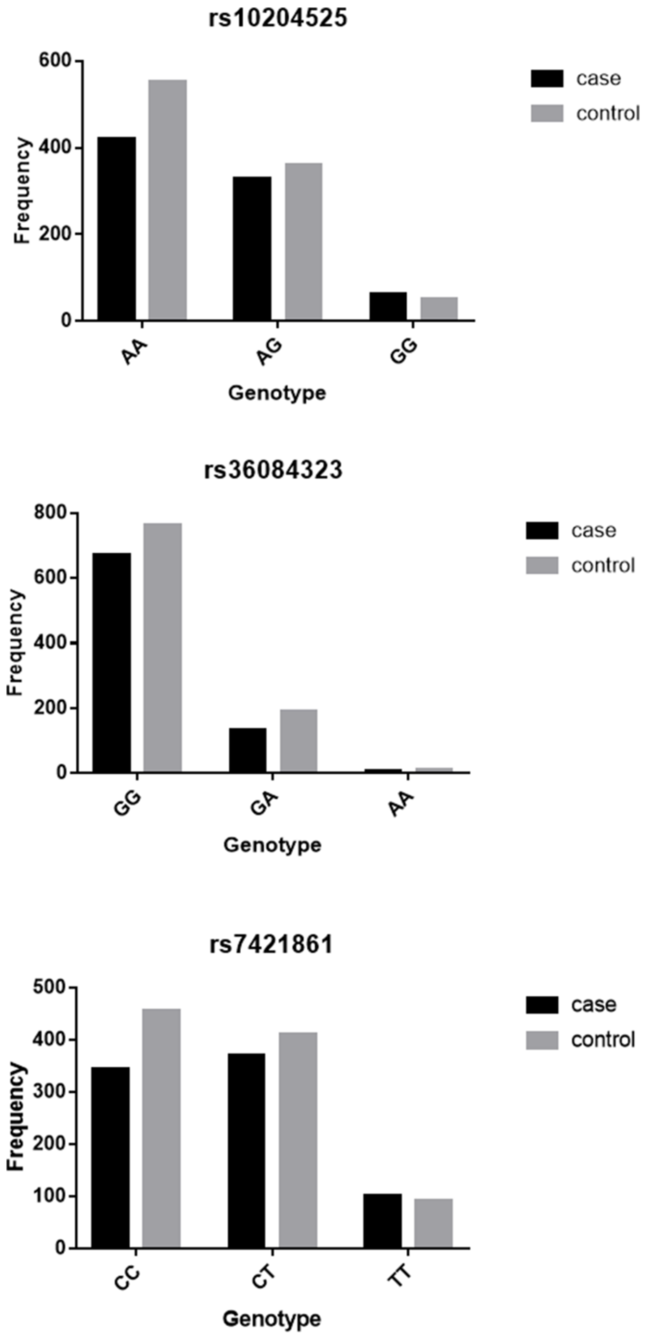

Supplementary Figure 1. The genotype numbers of rs10204525, rs7421861, and rs36084323 polymorphisms among esophageal cancer patients and controls.

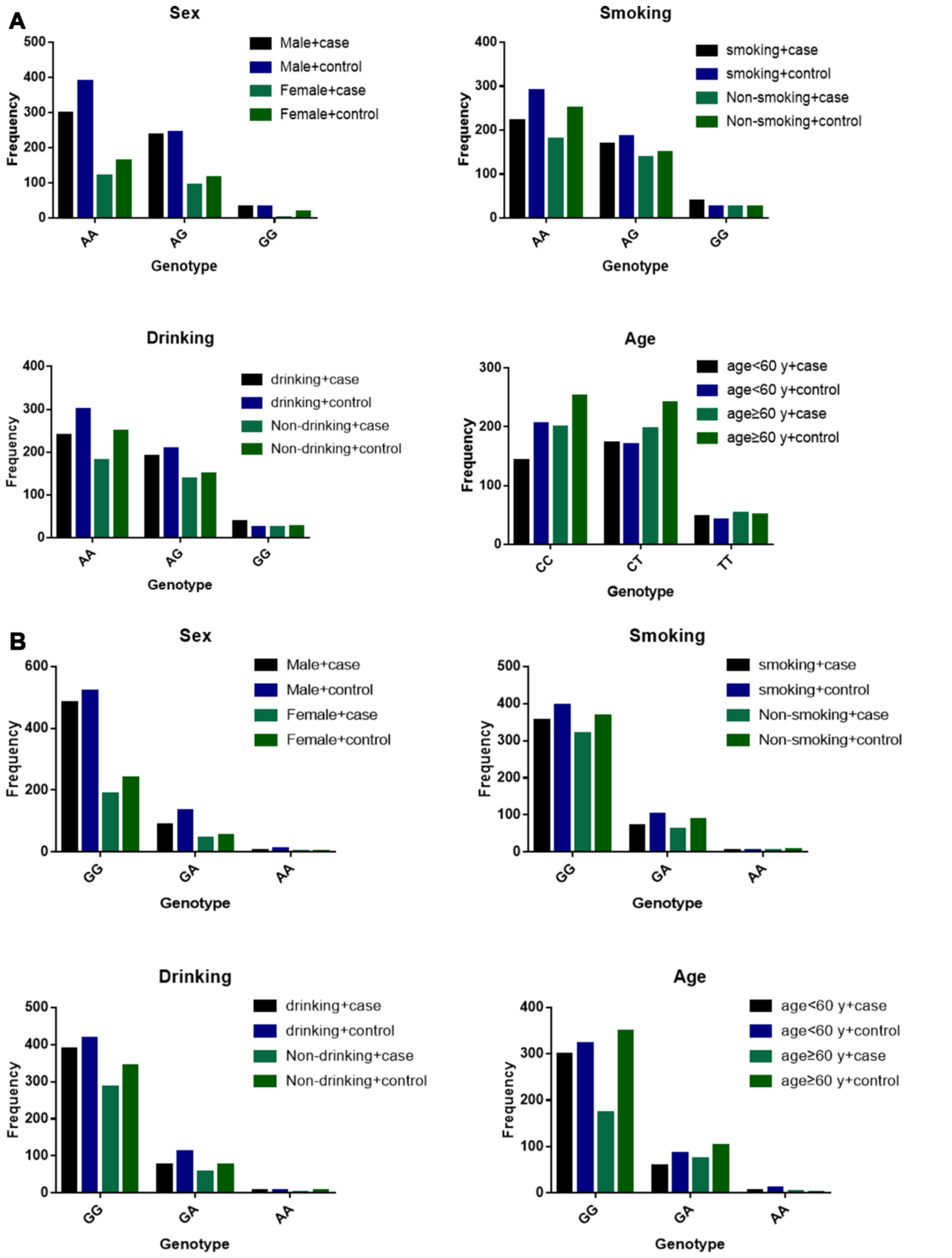
A
Sex
Male+case
Male+control
Female+case
Female+control
Frequency
Genotype
AA
AG
GG
Smoking
smoking+case
smoking+control
Non-smoking+case
Non-smoking+control
Drinking
drinking+case
drinking+control
Non-drinking+case
Non-drinking+control
Age
age<60 y+case
age<60 y+control
age≥60 y+case
age≥60 y+control
CC
CT
TT
B
Sex
Smoking
Drinking
Age
GG
GA
AA

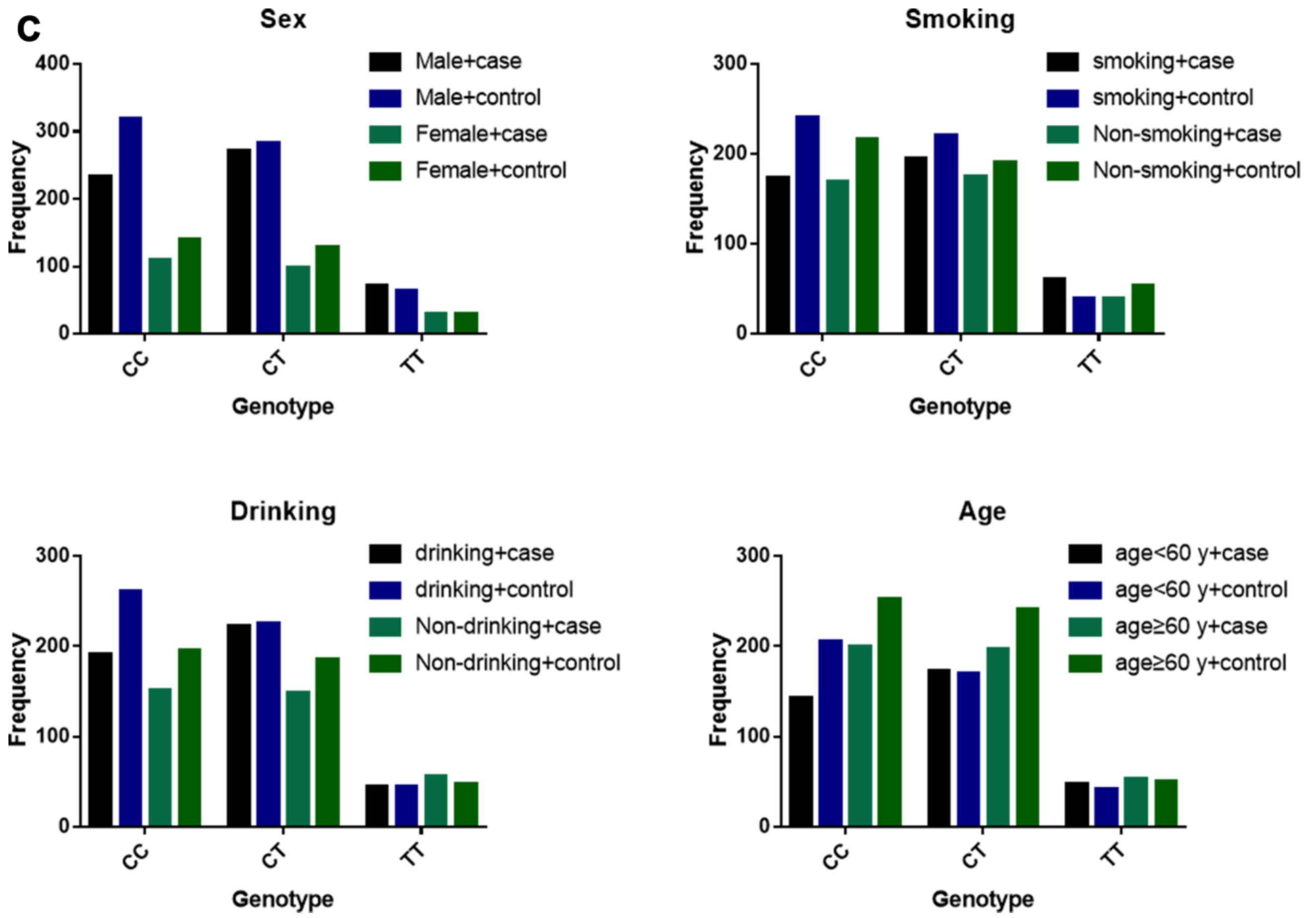

Supplementary Figure 2. The genotype numbers among different subgroups regarding (**A**) rs10204525 polymorphism, (**B**) rs36084323 polymorphism, and (**C**) rs7421861 polymorphism.
