# Supplementary material for: PD-1 gene rs10204525 and rs7421861 polymorphisms are associated with increased risk and clinical features of esophageal cancer in a Chinese Han population
Source: Aging (Albany NY). 2020 Feb 21;12(4):3771–90. doi: 10.18632/aging.102845 (PMC7066885; doi:10.18632/aging.102845)
Supplement: Supplementary Tables [file aging-12-102845-s001..pdf]

SUPPLEMENTARY TABLES

Supplementary Table 1. Genotype frequencies of PD-1 gene polymorphisms in patients with esophageal squamous cell carcinoma and controls.

Models	Genotype	Case (n, %)[a]	Control (n, %)[a]	OR (95% CI)	*P*-value	OR (95% CI)[b]	*P*-value[b]
**rs10204525**							
wild	AA	404 (51.2%)	551 (57.4%)	1.0 (reference)		1.0 (reference)	
Heterozygote	AG	316 (40.1%)	359 (37.4%)	1.20(0.99,1.46)	0.071	1.20(0.99,1.47)	0.068
Homozygote	GG	69 (8.7%)	50 (5.2%)	**1.64(1.10,2.43)**	**0.015**	**1.65(1.11,2.45)**	**0.013**
Dominant	AA	404 (51.2%)	551 (57.4%)	1.0 (reference)		1.0 (reference)	
	GG+AG	385 (48.8%)	409 (42.6%)	**1.25(1.04,1.52)**	**0.020**	**1.26(1.04,1.52)**	**0.018**
Recessive	AG+AA	720 (91.3%)	910 (94.8%)	1.0 (reference)		1.0 (reference)	
	GG	69 (8.7%)	50 (5.2%)	**1.52(1.03,2.24)**	**0.035**	**1.53(1.04,2.26)**	**0.032**
Allele	A	1124 (71.2%)	1461 (76.1%)	1.0 (reference)			
	G	454 (28.8%)	459 (23.9%)	**1.29(1.11,1.50)**	**0.001**		
**Rs36084323**							
wild	GG	645 (82.6%)	761 (79.2%)	1.0 (reference)		1.0 (reference)	
Heterozygote	GA	128 (16.4%)	188 (19.6%)	0.80(0.63,1.03)	0.083	0.80(0.63,1.03)	0.082
Homozygote	AA	8 (1.0%)	12 (1.2%)	0.79(0.32,1.94)	0.601	0.77(0.31,1.90)	0.569
Dominant	GG	645 (82.6%)	761 (79.2%)	1.0 (reference)		1.0 (reference)	
	AA+GA	136 (17.4%)	200 (20.8%)	0.80(0.63,1.02)	0.074	0.80(0.63,1.02)	0.071
Recessive	GA+GG	773 (99.0%)	949 (98.8%)	1.0 (reference)		1.0 (reference)	
	AA	8 (1.0%)	12 (1.2%)	0.82(0.33,2.01)	0.663	0.80(0.33,1.98)	0.630
Allele	G	1418 (90.8%)	1710 (89.0%)	1.0 (reference)			
	A	144 (9.2%)	212 (11.0%)	0.82(0.66,1.02)	0.080		
**rs7421861**							
wild	CC	329 (42.1%)	457 (47.6%)	1.0 (reference)		1.0 (reference)	
Heterozygote	CT	355 (45.5%)	411 (42.8%)	1.20(0.98,1.47)	0.075	1.20(0.98,1.46)	0.081
Homozygote	TT	97 (12.4%)	92 (9.6%)	**1.47(1.07,2.01)**	**0.020**	**1.47(1.07,2.02)**	**0.019**
Dominant	CC	329 (42.1%)	457 (47.6%)	1.0 (reference)		1.0 (reference)	
	TT+CT	452 (57.9%)	503 (52.4%)	**1.25(1.03,1.51)**	**0.022**	**1.25(1.03,1.51)**	**0.024**
Recessive	CT+CC	684 (87.6%)	868 (90.4%)	1.0 (reference)		1.0 (reference)	
	TT	97 (12.4%)	92 (9.6%)	1.34(0.99,1.81)	0.059	1.34(0.99,1.82)	0.056
Allele	C	1013 (64.9%)	1325 (69.0%)	1.0 (reference)			
	T	549 (35.1%)	595 (31.0%)	**1.21(1.05,1.39)**	**0.009**		

[a]The genotyping was successful in 780 cases and 960 controls for rs10204525; The genotyping was successful in 781 cases and 961 controls for rs36084323; The genotyping was successful in 781 cases and 960 controls for rs7421861.
Bold values are statistically significant (P <0.05).
[b]Adjust for sex, age, smoking and drinking.

Supplementary Table 2. Genotype frequencies of PD-1 gene polymorphisms in patients with other pathological types of esophageal cancer (not including esophageal squamous cell carcinoma) and controls.

Models	Genotype	Case (n, %)[a]	Control (n, %)[a]	OR (95% CI)	*P*-value	OR (95% CI)[b]	*P*-value[b]
**rs10204525**							
Wild	AA	16 (50.0%)	551 (57.4%)	1.0 (reference)		1.0 (reference)	
Heterozygote	AG	13 (40.6%)	359 (37.4%)	1.25(0.59,2.62)	0.561	1.22(0.58,2.56)	0.608
Homozygote	GG	3 (9.4%)	50 (5.2%)	2.07(0.58,7.33)	0.261	2.12(0.60,7.58)	0.246
Dominant	AA	16 (50.0%)	551 (57.4%)	1.0 (reference)		1.0 (reference)	
	GG+AG	16 (50.0%)	409 (42.6%)	1.35(0.67,2.73)	0.407	1.32(0.65,2.68)	0.439
Recessive	AG+AA	29 (90.6%)	910 (94.8%)	1.0 (reference)		1.0 (reference)	
	GG	3 (9.4%)	50 (5.2%)	1.88(0.56,6.39)	0.310	1.96(0.57,6.67)	0.284
Allele	A	45 (70.3%)	1461 (76.1%)	1.0 (reference)			
	G	19 (29.7%)	459 (23.9%)	1.34(0.78,2.32)	0.289		
**rs36084323**							
Wild	GG	28 (87.5%)	761 (79.2%)	1.0 (reference)		1.0 (reference)	
Heterozygote	GA	4 (12.5%)	188 (19.6%)	0.58(0.20,1.67)	0.311	0.56(0.20,1.63)	0.290
Homozygote	AA	0 (0%)	12 (1.2%)	N/A		N/A	
Dominant	GG	28 (87.5%)	761 (79.2%)	1.0 (reference)		1.0 (reference)	
	AA+GA	4 (12.5%)	200 (20.8%)	0.54(0.19,1.57)	0.259	0.53(0.18,1.52)	0.237
Recessive	GA+GG	32 (100.0%)	949 (98.8%)	1.0 (reference)		1.0 (reference)	
	AA	0 (0%)	12 (1.2%)	N/A		N/A	
Allele	G	60 (93.8%)	1710 (89.0%)	1.0 (reference)			
	A	4 (6.3%)	212 (11.0%)	0.37(0.13,1.02)	0.055		
**rs7421861**							
Wild	CC	14 (43.8%)	457 (47.6%)	1.0 (reference)		1.0 (reference)	
Heterozygote	CT	15 (46.9%)	411 (42.8%)	1.19(0.57,2.50)	0.643	1.24(0.59,2.60)	0.126
Homozygote	TT	3 (9.4%)	92 (9.6%)	1.06(0.30,3.78)	0.923	1.07(0.30,3.80)	0.576
Dominant	CC	14 (43.8%)	457 (47.6%)	1.0 (reference)		1.0 (reference)	
	TT+CT	18 (56.3%)	503 (52.4%)	1.17(0.57,2.38)	0.668	1.20(0.59,2.46)	0.609
Recessive	CT+CC	29 (90.6%)	868 (90.4%)	1.0 (reference)		1.0 (reference)	
	TT	3 (9.4%)	92 (9.6%)	0.98(0.29,3.27)	0.969	0.96(0.29,3.23)	0.949
Allele	C	43 (67.2%)	1325 (69.0%)	1.0 (reference)			
	T	21 (32.8%)	595 (31.0%)	1.08(0.64,1.85)	0.757		

[a]The genotyping was successful in 32 cases and 960 controls.
Bold values are statistically significant (P <0.05).
[b]Adjust for sex, age, smoking and drinking.
